# Collaboration of general practitioners and exercise providers in promotion of physical activity a written survey among general practitioners

**DOI:** 10.1186/s12875-015-0316-8

**Published:** 2015-08-06

**Authors:** C. J. Leemrijse, D. H. de Bakker, L. Ooms, C. Veenhof

**Affiliations:** Netherlands Institute for Health Services Research (NIVEL), Department of Local Organization of Care, PO Box 1568, 3500 BN Utrecht, The Netherlands; Tilburg University Tranzo, Tilburg School of Social and Behavioral Sciences, PO Box 90153, 5000 LE Tilburg, The Netherlands; Stichting Onbeperkt Sportief, PO Box 200, 3980 CE Bunnik, The Netherlands; Department of Rehabilitation, Nursing Science & Sport, University Medical Center Utrecht, Brain Center Rudolf Magnus, PO Box 85500, 3508 GA Utrecht, The Netherlands

## Abstract

**Background:**

General practitioners have an ideal position to motivate inactive patients to increase their physical activity. Most patients are able to exercise in regular local facilities outside the health care setting. The purpose of this study was to get insight into general practitioners perceptions and current practices regarding referral of patients to local exercise facilities. Furthermore, collaboration with exercise providers in the community was investigated, and motivators and barriers for referral.

**Methods:**

A written questionnaire sent to a representative random sample of 800 Dutch general practitioners. Descriptive statistics and Chi^2^ tests were used.

**Results:**

All responding general practitioners (340) recommend their patients to take more exercise when necessary and 87 % say to refer patients sometimes. Limited motivation of the patient (44 %) and reduced health status (34 %) are the most mentioned barriers for advising patients to increase physical activity. When referred, most patients are send to a physical therapist (69 %) but also local exercise facilities were mentioned (54 %). The most important barrier for referring patients to local exercise activities are patients limited financial possibilities (46 %). Restricted knowledge of local exercise- or sport facilities was an additional barrier (19 %). There is little structural collaboration between general practitioners and exercise providers, but when collaboration exists general practitioners refer more often. Positive experiences of patients (67 %), affordable offers (59 %) and information of local exercise facilities (46 %) are seen as important promoting factors for referral. Although 32 % of the general practitioners think that good collaboration would be stimulating, regular meetings with sports and exercise providers were considered the least important for increasing referral (3 %).

**Conclusions:**

Dutch physicians have a positive attitude towards stimulating physical activity but referral to local exercise facilities is low. Referral is partly hindered by restricted knowledge of local exercise facilities. Although general practitioners think that collaboration is important for physical activity promotion, it should not cost them much extra time. A coordinator with knowledge of the local situation can facilitate contacts between GP practices and sports providers.

## Background

The benefits of regular physical activity on health and quality of life are widely recognized. Physical activity reduces the risk of diseases like cardiovascular diseases, diabetes, osteoporosis, depression, some types of cancer and there is evidence that physical activity has protective effects for dementia risk [1–9]. Furthermore, physical activity contributes to one’s overall physical and mental wellbeing. Despite all known benefits, large groups of people are insufficiently active and inactivity is an important public health problem accounting for substantial healthcare costs [10–12].

In the Western world, mainly people with a lower socioeconomic status, people who are overweight, people with (an increased risk of) chronic conditions, and elderly are insufficiently active [13]. Specifically for those groups exercise could have positive health effects, but apparently they need extra support to start and maintain physical activity. The primary health care setting may offer a chance to reach these groups. In the Netherlands, every person is registered in one general practice. Although most practices are small (2–5 GPs), there is a trend to larger practices including other disciplines such as physical therapists (PTs), practice nurses and psychologists. The GP is the gatekeeper to hospital and specialist care. Practice nurses are employed to perform check-ups for the chronically ill and guide them in medication use and life style.

Nearly 80 % of all citizens visit their General Practitioner (GP) at least once a year, so GPs can identify patients who are insufficiently active and have, or are at risk for inactivity related health problems. Furthermore, GPs are a valued source of physical activity information, especially for elderly, those with chronic disease and the insufficiently active [14, 15]. Thus GPs (and subsequently the practice nurse) have a unique position to discuss the health benefits of regular physical activity with these patients and to motivate them to start with physical activities. Physical activity can of course be performed non-organised but compliance may be difficult, especially for people who are not intrinsically motivated to exercise. Therefore, to integrate exercising as a part of daily life, it should preferably take place in regular local exercise facilities under guidance from physical activity professionals, but outside the health care setting.

The procedure in which a GP identifies and refers sedentary patients with lifestyle related health problems to an exercise facility in the community is a common model of ‘exercise referral’. In the UK several exercise referral schemes are developed since the nineties, as well as in Spain and the Scandinavian countries. There is evidence that referral schemes may increase physical activity of sedentary people, but the effects were small, partly due to the diversity in the nature and quality of included exercise schemes [16–20].

In the Netherlands, exercise referral schemes are not widely used in daily practice. In 2010 the BeweegKuur programme (BK), which can be considered an example of exercise referral, was implemented in a restricted area [21]. In the BK, GPs referred patients to a lifestyle advisor who guided them towards increase of physical activity, either independently or (initially) under supervision of the physical therapist (PT). The main goal of the BK was a permanent increase of physical activity outside the health care setting. Collaboration between health care professionals and exercise providers was pursued. However, evaluation revealed that transfer from exercising under supervision to local exercise facilities was limited, and collaboration between health care professionals and physical activity providers was not widely spread yet [22]. Due to loss of financing the BK was never implemented nationally and collaboration between health care professionals and exercise providers must be established at local level.

Currently it is unknown to what extent Dutch GPs refer their patients to exercise facilities in the community and what factors would stimulate or hinder referral. We presume that referral by primary health care professionals to local exercise facilities will be enhanced when health professionals and local exercise providers know each other and cooperate in the promotion of physical activity [22]. The question raises however, to what extent GPs are willing to collaborate with local exercise providers. This study aims to get insight into GPs perceptions and current practices regarding referral of patients to community exercise facilities outside the health care setting. Furthermore, existing collaboration with exercise providers was investigated as well as motivators and barriers for referral outside the health care setting.

## Methods

A paper questionnaire was developed, based on (inter) national literature concerning (perceived stimulating factors and barriers in) the promotion of physical activity by GPs, and on literature regarding experiences with formal alliances on health promotion [23–33]. A concept was reviewed and complemented by experts from the Netherlands Institute for Sports and Exercise (NISB).

The questionnaire (Appendix 1) aimed to investigate:

- the perceived role of GPs regarding stimulating physical activity
- whether GPs referred patients to a physical therapist (inside the health care setting), or to a sports/health club or another exercise facility in the community. Hereby, ‘referral’ was distinguished from the more open ended ‘advising’ or ‘recommending’, but not restricted to a written ‘prescription’.
- barriers and motivators for referral outside the health care setting
- involvement of GPs in structural collaboration with local sports or healthclubs or other exercise providers to promote physical activity.

The questionnaire was sent to a representative random sample of 800 Dutch general practitioners. This sample was taken from the database of NIVEL (Netherlands Institute of Health Services Research) containing all practising Dutch GPs. After two weeks a reminder was sent to non-respondents.

### Statistical analysis

Descriptive statistics were performed. To compare results from GPs involved in formal alliances to promote physical activity and GPs who were not, Chi^2^ analyses were used (with a significance level of 0.05). The statistical package STATA 13.0 was used.

### Ethical considerations

As this study did not impose any interventions or actions, Dutch legislation does not require its approval by an ethic committee (under the Medical Reaearch Involving Human Subjects Act; http://www.ccmo.nl). Respondents received a letter informing them about the aim of the study. Participation was voluntary and responses anonymous.

## Results

340 questionairres were completed, leading to a net response of 43 %. Five questionnaires were returned unopened because of changed address (*n* = 3), closing of the practice (*n* = 1) or lack of interest (*n* = 1). Of the respondents (200 male and 140 female) 29 % were younger than 40 years and 30 % were 55 years or older. 159 GPs (47 %) worked alone, while 181 practised with colleague GPs. Respondents formed a representative sample of the study sample considering age, sex, form of practice (single or group) and degree of urbanisation. Compared to the whole Dutch population of GP’s, respondents were slightly younger (1,5 years) and were more often working alone (47 versus 28 %).

### Recommending more physical activity

Half of the GPs thought that they had an important role in stimulating physical activity, while the other half considered their role present but ‘limited’. All GPs said to recommend their patients to take more physical exercise. This advice was given when more exercise was relevant for the actual health problem for which the patient consulted the GP (mentioned by 79 % of the GPs), or when increasing physical activity was relevant for the patients general health status, irrespective of the actual health problem (mentioned by 48 %). Limited motivation of the patient (44 %) and patients’reduced health status (34 %) were the most mentioned barriers for advising patients to increase their physical activity (Table 1).

**Table 1:** Reasons for (not) giving physical activity advices

Reasons for giving physical activity advices	%
When relevant for the actual health problem	78.5
To specific groups of patients (such as people with (increased risk for) chronic diseases, e.g. DM)	48.5
When relevant for patients general health status (irrespective the actual health problem)	47.7
When patients ask for advise	16.5
Reasons for not giving physical activity advices	%
Patients limited motivation for increasing physical activity	44.4
Patients reduced health status	34.1
Lack of time (of the GP)	26.2
Patients cultural background/family situation/living conditions	15.9
Don’t think of giving advises	11.2

### Referral to an exercise facility

Of all GPs, 13 % never actually referred any patient to a specific physical activity facility in the neighborhood, but gave non-committal advice only. The other GPs not only gave informal advices to their patients to take more physical exercise, but also specifically referred them sometimes. Although most patients were referred to a PT within the health care setting (69 within and 34 % outside the GP-practice), GPs also mentioned to refer to a fitness centre (54 %) or to another local exercise facility (37 %). Mentioned facilities were specific (community) programs for elderly, specific programmes for people with (increased risk for) chronic diseases, and clubs for (Nordic) walking, running, swimming, or cycling. However, the actual number of referrals to a local fitness club or to other organized local activities is low. Most GPs estimated that 20 % or less of their patients who should increase their physical activity for health reasons were actually referred (Table 2).

**Table 2:** Percentage of GPs referring to a local exercise facility

Referral by GPs to a specific physical activity facility	%
Physical therapist outside the practice	68.5
Fitness centre	54.1
Another specific local activity or sports club (no fitness)	36.5
Physical therapist within the own practice	33.5
Practice nurse/lifestyle counsellor within or outside the practice	29.4

#### Motives and barriers for referral to a local exercise facility outside the health care setting

**Fig. 1:**
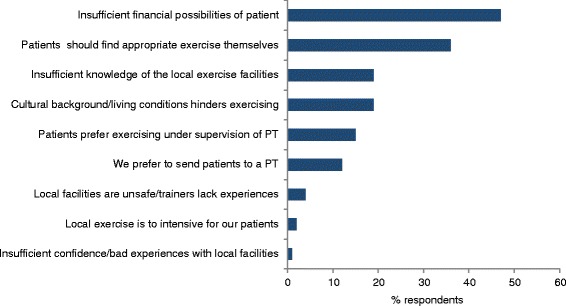
Reasons given by GPs for not referring patients to a local exercise facility, in percentages

Almost half of the GPs (49 %) thought that giving a specific and directed recommendation is more effective to stimulates patients to start with physical activity, compared to an open ended advice. Moreover, GPs referred to a local exercise facility because they thought that physical activity should take place outside the health care setting (mentioned by 44 %), and because they believed that there were good and accessible exercise facilities in the neighborhood (36 %). Most important barrier for referring patients were limited financial possibilities of patients (mentioned by 46 %). Restricted knowledge of the local exercise facilities was an additional reason for not referring patients (19 %) (Fig. 1).

#### Facilitators for referring more patients to local sports clubs or physical activity facilities

For GPs, positive experiences of patients would be the most important promoting factor for referring more patients to a specific sports or exercise facility (67 %), followed by an affordable offer (mentioned by 59 %), and a website with information of local facilities (46 %). Although one third of the GPs mentioned good collaboration as a stimulating factor for referring more patients to movement facilities, regular meetings of health professionals and sports providers (3 %) and exchange of information about the (activity) progress of patients (7 %), were considered the least stimulation for increasing referral to sports facilities in the community (Table 3).

**Table 3:** Facilitators for referring more patients to local sports clubs or physical activity facilities in the community

Facilitators	Respondents (%)
Positive experiences or effects for patients	66.5
Affordable physical exercise and sport facilities	58.8
Website with local sports and physical activity facilities	45.9
Flyers for patients with local sports and physical activity facilities	35.3
Exercise providers adequately trained in supporting inactive people^a^	33.8
Good collaboration with trainers of sports and exercise facilities	32.4
Introductory lessons against reduced price	30.0
Specific group programs for the target population	29.4
Reimbursement of physical exercise	22.4
Introductory meeting with local sports and exercise facilities	18.5
Financing of collaboration between GP and local exercise providers	18.5
Exchange of information about patients between trainers and GPs	6.8
Regular meetings of GPs and local exercise providers	2.7

^a^medical and or psychological support

### Formal collaboration between primary health care professionals and exercise providers

Only 17 % of the GPs (*n* = 57) participated in a formal alliance for stimulating physical activity. The most mentioned partners were other (allied) health care professionals such as PTs (93 %), colleague GPs (54 %) and practice nurses (51 %). Also local fitness centres (28 %) and life style counsellors (21 %) were frequently involved in collaboration, but other local exercise providers were hardly mentioned (11 %).

The main reason for not participating in a formal alliance was the fact that GPs thought that there was no such alliance in their neighborhood (65 %). One third of the respondents had never thought of participating. Almost three quarter of the GPs would be interested in participating in a local network for stimulating physical activity if this was available.

#### Differences in referral between GPs in a formal alliance for stimulating physical activity and GPs who are not

**Fig. 2:**
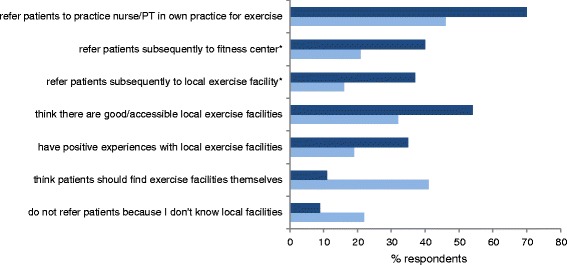
Differences between GPs participating in alliance for stimulating physical activity and GPs who are not

GPs participating in a formal alliance (*n* = 57) significantly more often said to refer patients to a practice nurse (*n* = 21; 37 %) or to a physical therapist (*n* = 25; 46 %) within their own practice to increase physical activity, compared to GPs who were not involved in an alliance. Sixty-three of these latter GPs (23 %) refer patients to a practice nurse and 86 (32 %) to a physical therapist. Additionally, the GPs participating in a formal alliance more often refer patients to a local fitness centre (*n* = 23; 40 versus *n* = 56; 21 % of the other GPs) or a specific exercise facility (*n* = 21; 37 versus *n* = 42; 16 %) after initial contact with the nurse practitioner or physical therapist. GPs not participating in a formal alliance were less informed about local exercise facilities and mentioned this more often as a reason for omitting referral (*n* = 59; 22 %) versus *n* = 5; 9 %) (Fig. 2).

## Discussion

Half of the GPs participating in the study believe they have an important role in stimulating their patients’ physical activity and none of them feels that this beyond his or her professional responsibility. These findings are in agreement with earlier studies in North America, UK and Europe, revealing that physicians had a positive attitude towards promoting physical activity [15, 34–37]. A positive attitude however, does not guarantee that stimulating physical activity is common practice. GPs estimated that 20 % or less of their patients who should increase their physical activity for health reasons were actually referred.

### Barriers in advising and referral to local exercise facilities

In the present study, the most frequently mentioned barrier for giving advice to increase physical activity is lack of motivation of patients. This seems to be a strange argument since especially patients who are not intrinsically motivated are insufficiently active and need stimulation from their GP. A study of Smidt et al. showed that being motivated by a GP is important for patients and provides a strong incentive to exercise [38]. However, GPs see around 30 patients per day and may have insufficient time to discuss such a difficult issue as increasing physical activity with patients who they expect to be less motivated. Referral of these patients to a practice nurse can be good alternative. Nurse practioners are trained in supporting people with chronic conditions for whom changing lifestyle is also often important. If GPs value exercise as an important part of treatment and take the initiative to raise the health benefits of physical activity with their patients, the practice nurse may be better able to pursue also less motivated patients to take more exercise. When patients have complex health problems also PTs may take such a motivational role, as long as the intention is transfer to local exercise facilities as soon as possible.

Another important barrier for stimulating more physical activity were limited health conditions. For some patients it may indeed be recommended to start exercising under supervision of a PT, but there may also be possibilities to participate in a specific adapted programme of physical activity, such as special swimming activities for people with chronic conditions or gym for the elderly. However, these specific programmes must be known to health professionals before they can suggest them to their patients. In this study, GPs reported to be little informed about local exercise facilities, illustrating the need of acquaintance and collaboration with local exercise providers.

More than half of the GPs sometimes actively refer their patients to a specific local exercise facility. The most frequently mentioned barrier for referring patients to a local movement facility were limited financial possibilities of the patient. This is in accordance with Franco et al. (2015), found 32 studies in which costs associated with physical activity programmes were considered a major barrier to participation [39]. However, other studies found that reducing the price of sports or other movement facilities did not result in higher participation levels [40], and costs might be an easy excuse to mask the real reason for being insufficiently active, that is limited motivation. Lack of financial possibilities can of course be a real problem for some people and development of affordable local movement facilities is important. Additionally, insurance companies might consider reimbursement of movement programmes for vulnerable groups. GP’s could of course also stimulate patients to exercise non-organised (e.g. walking, cycling). However, for people who are not used to be physically active this may be more difficult to maintain.

Many GPs are of the opinion that physical activity should be taking place outside the health care setting whenever possible. However, GPs also often refer patients to the PT to increase their level of physical activity. Many people in the Netherlands are insured for physical therapy. It is of course possible that GPs are reluctant to send their patients to local exercise facilities such as fitness centres that costs them money. In fact there are no real objections for (starting) physical activity in the PTs practice, as long as patients pay for this service themselves when there is no (longer) medical need to exercise under supervision of the PT. However, in order to incorporate physical activity as a natural and permanent part of daily life, it should rather be experienced as a pleasant manner to spent ones leisure time than as a (temporal) medical treatment. Therefore, physical activity preferably takes place outside the healthcare setting.

#### Formal collaboration between health care professionals and sports- and exercise providers

Before designing the study, we assumed that referral was easier and more daily business when GPs personally know the exercise providers and cooperate with them in promotion of physical activity in the community. In our study, less than one fifth of the GPs are involved in a a formal collaboration network with other health care professionals and physical activity providers. GPs who are participating in a formal alliance, indeed more often referred patients from their practice to a local fitness centre or sports facility. Furthermore, these GPs are more positive about the physical activity facilities in the neighborhood.

The question is however, to what extent GPs are really willing to participate in a formal alliance. The majority of health professionals who are not involved in a network states to be interested in participating in such a network when available in their neighborhood. However, when stimulating factors for referring more patients to a local sports club are asked, other factors are prioritized. Positive experiences from patients and affordable physical activity facilities are the most frequently mentioned stimulants for directing more patients to a local movement facility. A good collaboration with sport- and physical activity providers is also seen as promotional, however regular meetings and consultations about patients progress have the least priority. Respondents prefer a single information meeting about the sports clubs and other physical activity facilities in the neighborhood and flyers or a website presenting the different possibilities. So, enhanced collaboration between health care professionals and exercise providers would be welcome, but should not cost much of extra time and energy.

#### Recommendations for collaboration

The results of this study were discussed in an expert meeting with representatives from National health care organizations, sports organizations and policy makers to formulate recommendations for collaboration. An important recommendation following from the results of the study is that collaboration between health care providers and exercise providers should be set up rather simple. Collaboration should be based on the specific (health) needs and opportunities of the community. This could be arranged by establishing contact with initially a small number of local exercise providers around the practice. GPs, but also practice nurses and exercise providers should be introduced to each other and GPs must be able to rely on sports supervisors who are suitably trained for providing exercises to relative inactive persons. When exercise providers are more aware of the needs and preferences of the (patient) population, they have the opportunity to adapt their exercise programs or develop specific activities for people with health problems. The number of structural meetings between GPs and sports providers should remain limited. Connections may be maintained by the practice nurse or (if present) a PT of the practice and a single representative of each local exercise facility. It is known from the literature that a coordinator with knowledge of the local situation can facilitate the contacts between GP practices and sports providers, particularly in the early stages of cooperation [41, 42]. This coordinator can theoretically be anybody in the community, from practice nurse to welfare worker or fitness trainer, as long as he or she has good connections with both health care professionals and physical activity providers.

#### Strengths and limitations of the study

A limitation of this study is that results are based on self report and actual figures on advising and referring patients to increase their level of physical activity are unknown. Although the respondents were representative for the study sample with respect to age, sex, form of practice and region, they may of course have a more positive attitude towards stimulating physical activity compared to non-respondents, and possibly compared to the whole population of Dutch practitioners. A response of 43 % is rather high in studies with GPs.

## Conclusions

Dutch physicians have a positive attitude towards stimulating physical activity but referral to local exercise facilities is low. Cooperation between health care professionals and sports- and exercise providers is still rare, but GPs involved in cooperation refer more patients for physical exercise.

GPs see collaboration as a stimulating factor for referral. However, collaboration should not cost too much of their time. A coordinator with knowledge of the local situation can facilitate contacts between GP practices and sports providers.

## Appendix 1

**Table 4:** Items from the questionnaire for general practitioners

1. Do you think GPs have a professional role in stimulating physical activity?
□ No
□ Yes, but a limited role
□ Yes, a (very) important role
2. Do you advice your patients sometimes to take more exercise?
□ No, never
□ Yes, when patients ask for advise
□ Yes, to specific groups of patients (such as people with (increased risk for) chronic diseases, e.g. DM)
□ Yes, when relevant for the actual health problem
□ Yes, when relevant for the general health status (irrespective the actual health problem)
3. What are the reasons not to advise patients to take more exercise?
□ I don’t think this is my professional task
□ I don’t think of giving advises
□ Lack of time
□ I have to little knowledge of (more) physical exercise
□ I think it’s inconvenient to start talking about more physical activity
□ I doubt the health effects of physical activity
□ Patients limited motivation for physical activity
□ Patients reduced health status
□ Patients cultural background/family situation/living conditions
□ Other, …..…
4. Do you refer your patients sometimes to take more exercise?
□ No, I give non-committal advice only
□ Yes, to a practice nurse /lifestyle counsellor within the practice
□ Yes, to a life style counsellor outside the practice
□ Yes, to a physical therapist within the practice
□ Yes, to a physical therapist outside the practice
□ Yes, to a local fitness centre in the in the community
□ Yes, to another exercise facility in the in the community (no fitness)
□ Yes, other…..
5. What are reasons for referring patients to a (organized) physical activity or fitness in the community?
□ Inapplicable (I never refer my patients)
□ Patients are more willing to respond to a direct referral
□ It is good to stimulate physical activity outside the health care setting
□ There are good and accessible exercise facilities in the community
□ There is a good cooperation with sports and exercise providers in the community
□ We have good experiences with sports and exercise facilities in the community
□ We do not have enough expertise on physical activity ourselves
□ Other…..
6. What are reasons for not referring patients to a (organized) physical activity or fitness in the community?
□ Patients themselves should find an appropriate exercise facility
□ We prefer to send patients to a PT for physical activity
□ Patients prefer exercising under supervision of the PT
□ Insufficient knowledge of local physical activity facilities
□ Insufficient confidence in local exercise facilities
□ Bad experiences with local physical activity facilities
□ Local exercise facilities are unsafe/
□ Trainers of local exercise facilities lack experiences with our group of patients
□ Community physical activity are to intensive for our patients
□ Patients insufficient financial possibilities to participate in organised physical activity
□ Cultural background/family or living conditions hindering physical activity
□ Other, …
7. What proportion of patients who should exercise more for health reasons is actually referred to (organized) physical activity or fitness in the community?
□ 0 %
□ 1-20 %
□ 21-40 %
□ 41-60 %
□ 61-80 %
□ 81-100 %
8. Are you involved in a formal collaboration or network with other health care providers and/or sport and exercise providers in the community to stimulate physical activity?
□ Yes
□ No
9. Who are involved in this network/collaboration?
□ Collegue GPs
□ Practice nurse
□ Physical therapist
□ Life style counselor
□ Fitnesscentre
□ Leisure centre/welfare organisation/home care
□ Patiënts organisation
□ Local exercise facilities (such as sportclubs, hiking- or cyclingclubs, swimming pools, dancing centres etc.
□ Municipal health organisation
□ Municipality
□ Insurance company
□ Other, ..…..
10. Why is the practice not involved in such a formal collaboration or network to stimulate physical activity?
□ We have no interest in such a collaboration
□ As far as I know there is no formal collaboration or network in the community
□ There is a formal collaboration but this does not function adequately
□ It never occurred to me to participate in such a formal collaboration
□ Other……
11. Would your practice be interested in participating in a formal network with health professionals and sports providers when this was available in the neighborhood?
□ No, we are not interested
□ No, because a lack of time
□ Yes, if there would be a good functioning network
□ Yes, but only if this would be financed
□ Yes, because…..….
12. What would be stimulating factors for your practice to refer more patients to local sports and exercise facilities?
□ None
□ Positive effects for my patients
□ Positive experiences for my patients
□ Introductory information meeting about possibilities to refer to local sports- and exercise facilities
□ Good collaboration with trainers from local sports- and exercise facilities
□ Regular meetings with health care professionals and local sports- and exercise providers
□ Trainers adequately trained (medically/psychologically) in supporting inactive people
□ Specific group programmes for the target population
□ Exchange of information about patients progress between trainers and GPs
□ Website with local sports and exercise facilities
□ Flyers for patients with local sports and exercise facilities
□ Introductory lessons against reduced price
□ Affordable sports- and exercise facilities
□ Reimbursement of physical activity
□ Structural financing of collaboration between GP and local exercise providers
□ Other, ….

## Abbreviations

GP: General Practioner
PT: Physical Therapist
